# Follicular lymphoma and marginal zone lymphoma: how many diseases?

**DOI:** 10.1007/s00428-022-03432-2

**Published:** 2022-11-17

**Authors:** Camille Laurent, James R. Cook, Tadashi Yoshino, Leticia Quintanilla-Martinez, Elaine S. Jaffe

**Affiliations:** 1 Department of Pathology, Toulouse University Hospital Center, Cancer Institute University of Toulouse-Oncopole, Toulouse, France; 2 Department of Laboratory Medicine, Robert J. Tomsich Institute of Pathology and Laboratory Medicine, Cleveland Clinic, Cleveland, OH USA; 3 Department of Pathology, Graduate School of Medicine Dentistry and Pharmaceutical Science, Okayama University, Okayama, Japan; 4 Institute of Pathology and Neuropathology, Eberhard Karls Univesity of Tübingen and Comprehensive Cancer Center, University Hospital Tübingen, Tuebingen, Germany; 5 National Cancer Institute, National Institutes of Health, Bethesda, MD USA

**Keywords:** Follicular lymphoma, Marginal zone lymphoma, Classification, Morphological variants and distinct entities, 2022 ICC, 5th WHO

## Abstract

Follicular lymphoma (FL) and marginal zone lymphoma (MZL) are indolent mature B-cell neoplasms with variable clinical presentation and distinct histopathologic features. Recent advances in the biology and molecular characteristics of these lymphomas have further expanded our understanding of the heterogeneous nature of these lymphomas, with increasing recognition of specific disease entities within the broader categories of FL and MZL. Here, we discuss the conclusions of the 2022 International Consensus Classification of Mature Lymphoid Neoplasms (2022 ICC) dealing with FL, and review differences with the proposed WHO 5th Edition classification. We review issues related to grading and alternative forms of FL especially those lacking the genetic hallmark of FL, the t(14;18) chromosomal alteration. Among them, t(14;18)-negative CD23^+^ follicle center lymphoma has been proposed by the 2022 ICC as a provisional entity. Other follicle center–derived lymphomas such as pediatric-type follicular lymphoma, testicular follicular lymphoma, primary cutaneous follicle center lymphoma, and large B-cell lymphoma with *IRF4* rearrangement are considered distinct entities separate from conventional FL. Importantly, large B-cell lymphoma with *IRF4* rearrangement introduced as a provisional entity in the WHO 2017 is upgraded to a definite entity in the 2022 ICC. We also discuss diagnostic strategies for recognition of MZLs including splenic MZL, extranodal MZL (MALT lymphoma), and primary nodal MZL. The importance of molecular studies in the distinction among marginal zone lymphoma subtypes is emphasized, as well as their value in the differential diagnosis with other B-cell lymphomas.

## Introduction

Follicular lymphoma (FL) and marginal zone lymphomas (MZL) are indolent mature B-cell neoplasms with variable clinicopathologic and genetic features, with current evidence supporting the conclusion that they include multiple disease entities. Since the publication of the WHO 2017 classification [1], recent findings have provided new insights to refine FL and MZL diagnoses based on clinical presentation as well as their histological and molecular features. Subsequently, alternative forms of FL including variants and distinct subtypes, especially those lacking *BCL2* rearrangement, have been recognized in the 2022 International Consensus Classification (ICC, Table 1) [2]. Some recommendations from ICC 2022 [2] dealing with ancillary studies for FL grading as well as for classifying some cytological/morphological variants or entities distinct from conventional FL have been delineated, and some differences with the 5th WHO classification [3] were also addressed. Finally, we further emphasize the increasing role of molecular studies in the differential diagnosis of some cases of FL and MZL.

**Table 1:** International Consensus Classification of follicular lymphomas, marginal zone lymphomas, and related entities

Follicular lymphoma
In situ follicular neoplasia
Duodenal-type follicular lymphoma
BCL2-R-negative, CD23-positive follicle center lymphoma*
Pediatric-type follicular lymphoma
Primary cutaneous follicle center lymphoma
Testicular follicular lymphoma*
Large B-cell lymphoma with *IRF4* rearrangement*
Splenic marginal zone lymphoma
Extranodal marginal zone lymphoma of mucosa-associated lymphoid tissue (MALT lymphoma)
Primary cutaneous marginal zone lymphoproliferative disorder*
Nodal marginal zone lymphoma
*Pediatric nodal marginal zone lymphoma*

Items in italics represent provisional entities

^*^Changes from the 2017 WHO classification

## Follicular lymphomas

FL is a B-cell neoplasm derived from germinal center (GC) cells that is largely composed of a mixture of cleaved centrocytes (CC) and non-cleaved centroblasts (CB). Conventional FL involves mainly nodal sites and is characterized by a clonal proliferation of follicle center cells harboring t(14;18) and exhibiting a follicular growth pattern. Other commonly involved sites include bone marrow, spleen, and gastrointestinal tract. Distinct FL forms have been described based upon age, anatomic sites, characteristic morphological features, and the presence or absence of the t(14;18) chromosomal alteration. Among them, early in situ lesions of FL have been recognized as indolent disorders closely related to conventional FL. The new category of t(14;18)-negative CD23^+^ follicle center lymphoma (FCL), which can have a follicular as well as a diffuse growth pattern, has been proposed by the 2022 ICC as a provisional entity [2]. Other variants of FL such as pediatric-type follicular lymphoma (PTFL), testicular follicular lymphoma (TFL), primary cutaneous follicle center lymphoma (PCFL), and large B-cell lymphoma with *IRF4* rearrangement (LBCL*-IRF4*) are considered as distinct entities separate from conventional FL in the 2022 ICC [2].

### Conventional follicular lymphoma

#### Morphological features

FL is characterized by effacement of normal lymph node architecture by closely packed neoplastic follicles with loss of polarization, absence of body macrophages, and attenuated mantle zones. The neoplastic cells are characteristically a mixture of CC and CB. The 3rd and 4th edition World Health Organization (WHO) classifications of lymphomas [1, 4] recommended FL grading based on the number of CB per high-power field (HPF): grade 1 < 5CB/HPF; grade 2 = 5 to 15 CB/HPF; and grade 3 or high-grade > 15 CB/HPF. Grade 3 is subdivided into grades 3A and 3B; the latter is assigned to FL cases, where neoplastic follicles are essentially composed of sheets of CB. Moreover, because grades 1 and 2 comprise a morphologic continuum and are both clinically indolent, the designation grade 1–2 (low-grade FL) was introduced and widely used [1, 4]. However, FL grading based on strict counting methods of CB and CC (cells that may exhibit variation in their cytological features) lacks reproducibility and its clinical impact, especially between grades 1, 2, and 3A, appears to be less significant in the era of rituximab. Therefore, the 5th edition of the WHO [3] proposes grading to be optional in FL, gathering grades 1, 2, and 3A as classic FL, whereas FL grade 3B was renamed as follicular large B-cell lymphoma (FLBL). Although, the 2022 ICC [2] recognizes the biological relationship between grades 1, 2, and 3A, and acknowledges some difficulties in FL grading, the consensus among clinicians was to retain histologic grading, in part to allow for investigation of the response of new agents. Whether patients with grade 3A have a more adverse prognosis [5, 6] or deserve different management remains debatable and needs to be re-evaluated in the future, given evolving non-cytotoxic therapeutic approaches [5–9]. Of note, some FLs are composed of neoplastic cells that are not classic CB but rather small-sized blasts/blastoid cells or large cleaved cells/large centrocytes that do not strictly fit the FL3A or FL3B 2017 WHO criteria [10]. Based on the challenges in distinguishing FL 3A and 3B, the 2022 ICC recommends that emphasis should be placed on ancillary studies in conjunction with morphological assessment. The demonstration of *BCL2* rearrangement (*BCL2*-*R*) and CD10 expression both favor grade 3A [2]. The 5th edition WHO recommends the name of FL with uncommon features (uFL) for these and other cases with variant morphology [3]. FL commonly has a follicular growth pattern sustained by a follicular dendritic cell (FDC) network. Sometimes more and less extended diffuse areas can be observed. Diagnostic reports traditionally identify the growth pattern, with designation as mainly follicular pattern (at least 75%); FL with follicular and diffuse pattern (25–75%); predominantly diffuse (< 25%); and totally diffuse (no follicles). Moreover, t(14;18)-negative CD23^+^ FCL, often having a diffuse growth pattern, should be also distinguished from conventional FL (see below). As recommended in the last WHO 2017 [1] and 2022 ICC [2], the presence of entirely or predominantly diffuse areas, in cases with high-grade cytology, will support a diagnosis of diffuse large B-cell lymphomas (DLBCL). However, treatment decisions in individual patients should not be based on pathology information alone but rather on integration of clinical and pathologic data [3].

In addition, several cytologic variants including FL with marginal zone B-cell differentiation (monocytoid-like cells), floral variant, or sclerosing variant (Castleman-like features) of FL have been described but are not separated from conventional FL. Other cytologic FL variants are less commonly seen.

#### Phenotype

FL cells express the pan B-lymphocyte antigens (CD19, CD20, CD22, CD79a, PAX-5) and IgM with or without IgD. Similar to normal GC cells, FL cells express CD10 and BCL6, usually stronger in the neoplastic follicles than in interfollicular areas. Moreover, a subset of FL especially grade 3B can be negative for CD10 and/or BCL6. In this context, other GC markers including GCET1, HGAL (GCET2), LMO2, and/or MEF2B should be performed to establish a GC phenotype and rule out other B-cell lymphoma diagnoses.

FL cells typically overexpress BCL2 as a result of the genetic hallmark of FL, t(14;18) *IGH::BCL2* translocation. Similar to CD10 staining, the frequency of BCL2 expression is higher in low-grade (85–90%) than in high-grade (50–70%) FL. Overall 10–15% of FL cases, especially grade 3B [10, 11], remain BCL2-negative due to the lack of *BCL2-R*. The variable expression of BCL2 may also be due to mutations in the *BCL2* gene that alter the epitopes recognized by some monoclonal antibodies used for diagnosis. In these “pseudo-negative” cases, other BCL2 antibodies and/or FISH for *BCL2*-*R* should be tested [12–14].

FL is commonly negative for IRF4/MUM1. However, it can be expressed in some cases, especially with grade 3B cytology. In these particular cases of IRF4/MUM1-positive FL grade 3B, *NOTCH1/2* mutations have been reported to be associated with poorer prognosis [15]. In addition, all IRF4/MUM1-positive FL with high-grade cytological features, especially grade 3B and uFL [2, 3], should be evaluated for *IRF4* alterations, especially in younger patients to rule out LBCL*-IRF4* (see below).

The follicular pattern of FL may be assessed by the identification of FDC networks that are positive for CD21, CD23, and/or CD35, usually absent in cases with diffuse areas. FL with grade 1–2 usually shows a lower Ki-67 proliferation index (PI) than grade 3. However, some FL 1–2 can exhibit a high PI within follicles. The significance of an increased proliferative rate within follicles is not established [16–18]. Assessment of PI using Ki67 staining can be difficult because its distribution is not uniform within follicles and its expression may be highly variable between follicular and interfollicular areas. Altogether, Ki67 staining can be specified in diagnostic reports, but still has uncertain clinical significance in isolation [19], and is not required for grading.

#### Genomics

##### FL with BCL2 rearrangement (*BCL2-R*)

The t(14;18) (q32;q21) or on rare occasions its variants t(2;18)(p12;q21) or t(18;22)(q21;q11) is the hallmark of conventional FL occurring in 85–90% of cases and leading to the overexpression of anti-apoptotic protein BCL2. Like BCL2 staining, the *BCL2-R* is more often observed in low-grade than in high-grade FL. However, t(14;18) can also be detected at a very low level in the peripheral blood of healthy adults (referred to as “FL-like cells”) indicating that the translocation alone is not sufficient for the development of FL, and additional genetic alterations are needed for full transformation. In fact, the acquisition of t(14;18) allows FL-like cells to iteratively re-enter GC and engage multiple cycles of somatic hypermutation (SHM), and class switch recombination (CSR) increasing the risk of accumulation of genomic instability. Mutations in epigenetic regulators and chromatin-remodeling genes are the most frequent in conventional FL [20]. Among them, chromatin-remodeling genes such as *KMT2D/MLL2*, *CREBBP*, and *EP300* mutations represent early drivers of conventional FL. Epigenetic dysregulation comprises gain-of-function mutations of *EZH2* that occurs in 25% of cases and may make them good candidates for EZH2 inhibition [21]. Other less frequent epigenetic modifier mutations are seen in *ARID1A, MEF2B,* and *KMT2C* genes. *BCL2* mutations are common due to AID activity. *TNFRSF14* mutations and deletions can be observed. In addition, other mutations involving cell signaling such as *STAT6, CARD11,* and *FOXO1* are less frequently seen. Genomic alterations in *TP53* or *CDKN2A and MYC* translocations are usually associated with high-grade features and/or risk of transformation [22]. According to the literature, MYC and TP53 IHC can be used as a screen for detecting genetic alterations among these genes but sensitivity and specificity vary between studies [23–27]. Moreover, the prognostic value of de novo FL-*BCL2R* with *MYC-R* is still controversial. Based on limited data available [28], de novo FL-*BCL2R*/*MYC-R* should not be included within the category of high-grade B-cell lymphoma with *MYC* and *BCL2* and/or *BCL6* rearrangements. At the moment, investigation for the presence of *MYC-R* or *TP53* mutations is not recommended on a routine basis [29]. In addition, other ancillary genomic studies for prognosis in FL, such as the M7-FLIPI, remain investigational [20, 29, 30].

##### FL without *BCL2* rearrangement (*BCL2-R*-negative)

*BCL2-R*-negative FL, representing 10–15% of FL cases, is heterogeneous, both genetically and clinically [31–33]. This group includes some conventional FL as well as alternative forms of FL that should be diagnosed separately. At least three groups are recognized: (1) FL with *BCL6-R* seems to share similar genetic alterations with conventional FL *BCL2-R*, but at different frequencies [33, 34]. Although studies on FL *BCL6-R* remain heterogeneous and difficult to compare with FL *BCL2-R*, such cases have been associated with more aggressive diseases [33, 34]. They show less frequent expression of CD10 and are often positive for MUM1/IRF4 [10, 33–36]. *NOTCH* mutations are reported in this group, suggesting overlap with nodal MZL [15]; 2) *BCL2-R*-negative and *BCL6-R*-negative cases often lack CD10 and CD23 expression, also raising the question of nodal MZL. In this context, NGS analysis may help in the differential diagnosis; 3) The new provisional entity *BCL2-R*-negative, CD23^+^ FCL (see below).

### Early lesions of follicular lymphoma

In situ* follicular neoplasia (ISFN)* is characterized by a monoclonal proliferation of B-cells carrying t(14;18) confined to the GC [37, 38]. Clinically, ISFN is an incidental finding with a low risk of progression to FL. ISFN is usually diagnosed incidentally in a reactive-looking lymph node with preserved nodal architecture, open sinuses, and well-defined GCs with intact mantle zones. BCL2 and CD10 are strongly expressed in the tumor B-cells (almost CC-like cells) confined to the GC. Proliferation is very low as demonstrated by MIB1.

*Duodenal-type FL (DFL)* is a neoplastic follicular proliferation containing CC-like cells harboring t(14;18) as FL. DFL is often discovered incidentally with a low risk of progression to systemic FL.

*Partial involvement of FL (PIFL)* is a partial destruction of nodal architecture by enlarged follicles with attenuated/disrupted mantle areas. Neoplastic follicles are similar to FL with atypical B-cells almost CC-like cells positive for t(14;18).

Besides the presence of *BCL2*-*R*, early FL lesions share similar genetic alterations as FL but at lower frequencies [39–41]. However, DFL is characterized by a chronic inflammation gene signature similar to that of mucosa-associated lymphoid tissue lymphoma (MALT lymphoma) [41, 42]. Interestingly, distinct early lesions or lesions occurring at different sites have been shown to be clonally related suggesting that early clonal cells can recirculate and reach different compartments of lymphoid tissues [41, 43]. Recently, Vogelsberg et al. [44] have demonstrated branched clonal evolution rather than a linear one between early FL lesions and manifest lymphomas, suggesting that ISFN and FL evolve from a common progenitor.

### Alternative forms of follicular lymphoma

#### ***BCL2-R-negative CD23***^+^***folliclecenter lymphoma (FCL)***

**Fig. 1:**
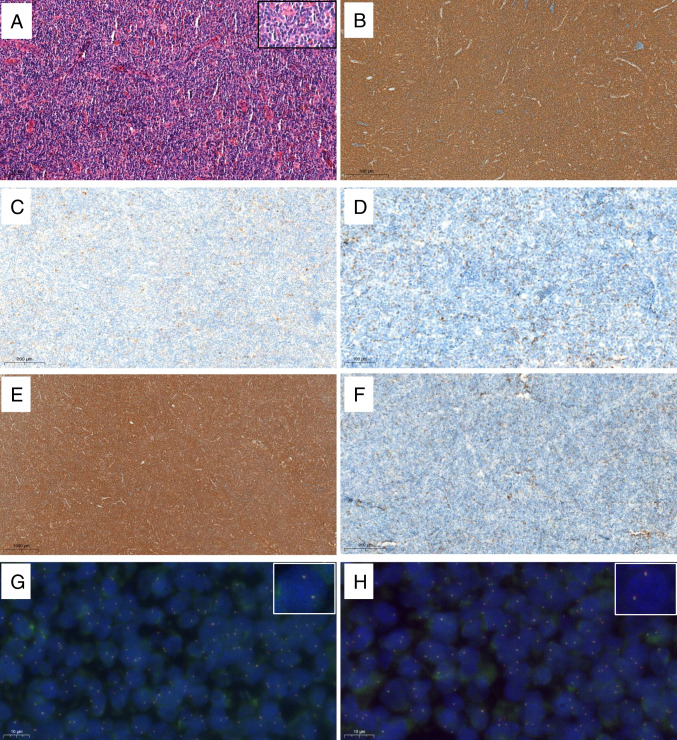
*BCL2*-R-negative CD23^+^ follicle center lymphoma with predominant diffuse growth pattern. Inguinal lymph node with effaced architecture by an atypical lymphoid infiltration with diffuse pattern (**A**, H&E). Tumors cells are CD20-positive (**B**), CD10-negative (**C**), BCL2-negative (**D**), and CD23-positive (**E**). CD21 shows no FDC meshwork (**F**). Interphase fluorescence in situ hybridization (FISH) using *BCL2* (**G**; inset × 6000) and *BCL6* (**H**; inset × 6000) break apart probes are negative

In 2009, Katzenberger et al. [45], described a diffuse variant of FL with unusual clinical and pathological features. Subsequently, it was found that these cases frequently carry *STAT6* mutations [31]. Further studies reported a close association of CD23 expression with *STAT6* mutation, features which correlated with localized disease. Moreover, it was appreciated that at least 30% of the cases were purely follicular [33]. Due to their characteristic, clinical, morphological, and molecular features, the 2022 ICC proposed recognizing these cases as a provisional entity [2]. The specific category of *BCL2-R*-negative CD23^+^ FCL is not recognized in the 5th WHO [3], although there is some overlap with the subtype identified as “diffuse variant.” *BCL2-R*-negative CD23^+ ^FCL is more frequent in women (M:F = 2:1), and often presents with inguinal involvement, but non-inguinal presentations including cervical, axillary, and even retroperitoneum can occur [31]. Patients usually present with low clinical stage. The characteristic histological features are illustrated in Fig. 1. Diagnostic criteria include the absence of the t(14;18), expression of CD23, and at least one GC marker. BCL2 staining is usually negative or weak positive. The molecular profile includes a high frequency of *STAT6* and *CREBBP* co-mutation as well as 1q gain and a recurrent loss 1p36 loss/*TNFRS14* abnormalities. The latter has been described at variable frequencies (30–97%) [33, 46]. As noted, CD23 expression is a helpful surrogate marker for the detection of *STAT6* mutations [33]. Interestingly, *BCL2-R*-negative CD23^+^ FCL without *STAT6* mutations carry *SOCS1* mutation, which is known to be upstream of STAT6 and thereby contributes to STAT6 activation [33].

#### Pediatric-type follicular lymphoma (PTFL)

PTFL was recognized as a definite entity in the WHO 2017 classification [1]. It occurs in children and adolescents, and has an excellent prognosis with conservative management. PTFL is usually characterized by an expanded and serpiginous follicular proliferation of monotonous CB, often with a starry sky pattern and moderate to high proliferation. Neoplastic cells express GC markers whereas BCL2 is typically negative. *BCL2-R*, *BCL6-R*, *MYC-R*, and *IRF4-R* are negative. The mutational profile is distinct from that observed in conventional FL with a high frequency of *MAP2K1* mutations and 1p36/*TNFRS14* alterations (30–70%) [47–49]. Additional mutations in *IRF8*, a tumor suppressor gene, are also common in PTFL (15–50%) [49–51]. Mutations in epigenetic modifiers are uncommon, in contrast to conventional FL. Among them, *KMT2D* is the most frequent mutated gene, observed in 16% but alterations in other genes such as *CREBBP*, *EP300*, *MEF2B*, and *EZH2* are rare. PTFL frequently shows evidence of marginal zone differentiation, and recent studies support the view that pediatric-type nodal marginal zone lymphoma (pNMZL) is part of the spectrum of PTFL, with both disorders having similar molecular profiles, clinical presentations, and outcomes [51]. Importantly, FISH testing and mutational profile are advised to rule out a diagnosis of conventional grade 3B FL as well as LBCL*-IRF4*, especially in young patients.

#### Primary cutaneous follicle center lymphoma (PCFL)

PCFL will be discussed elsewhere in this issue with other cutaneous lymphoproliferative disorders.

#### Testicular follicular lymphoma

Testicular follicular lymphoma occurs in children and is only rarely reported in adults. This variant has been recognized as a distinct form in 2022 ICC [2] but still considered as a subtype of FL in the 5th WHO [3]. It shares the same morphological and molecular profile as FL *BCL2-R*-negative with negative or weak expression of BCL2 and absent *BCL2*-*R*. Frequent *TNFRSF14* alterations, as seen in PCFL, and *MAPK* mutations, as observed in PTFL, have been reported [52] but further studies are needed to define the molecular landscape of this specific variant.

#### Large B-cell lymphoma with *IRF4* rearrangement (LBCL*-IRF4*)

**Fig. 2:**
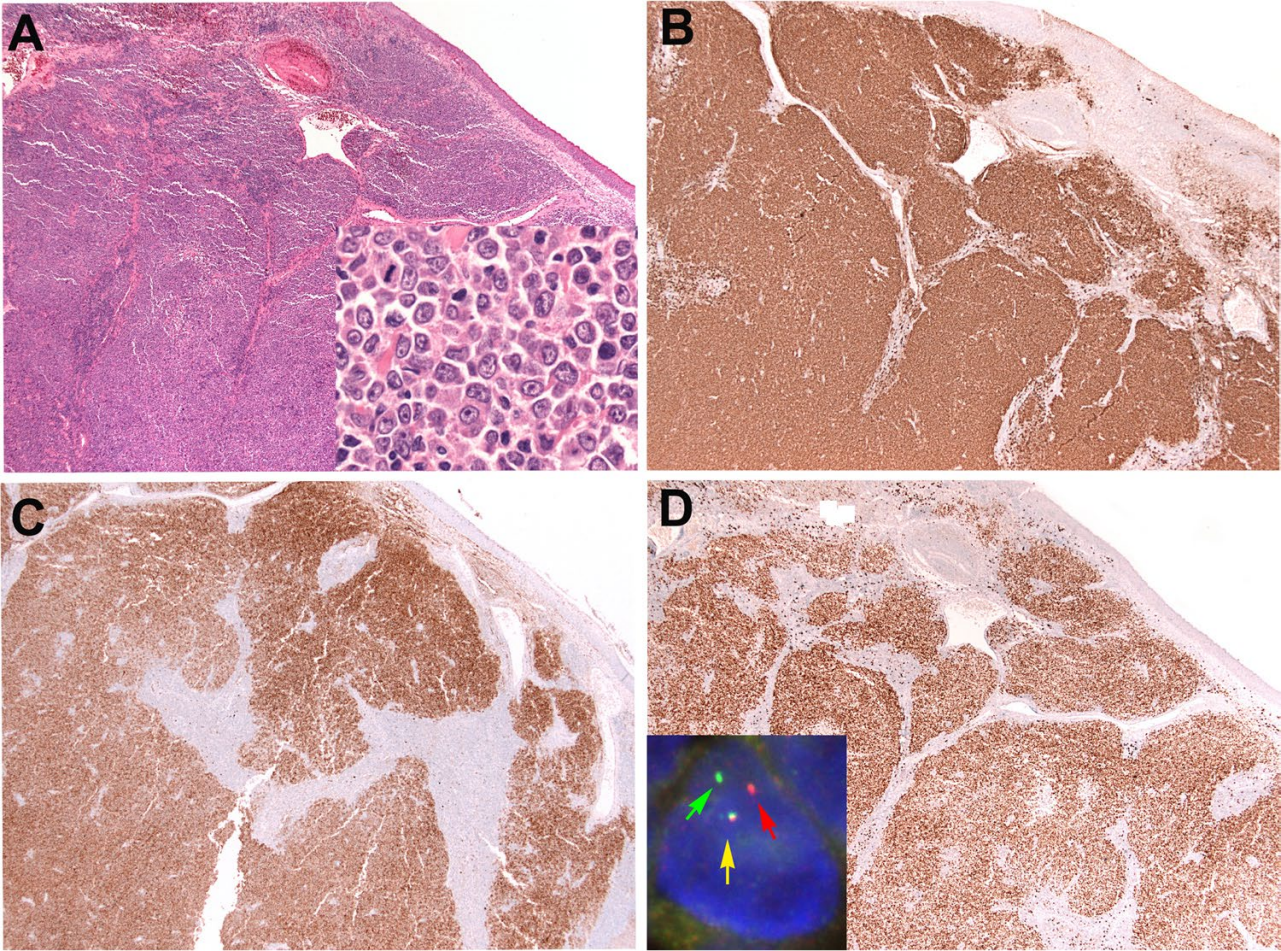
Large B-cell lymphoma with *IRF4* rearrangement. **A** H&E stain of a tonsil of a 37-year-old woman in clinical stage 1 disease at low magnification showing a vaguely nodular lymphoid proliferation (**A**, × 400; inset: centroblast-like cells comprise the dominant cell type). **B** The cells are CD20-positive. Note the vaguely nodular growth pattern. **C** CD10 is positive. **D** MUM1/IRF4 is strongly and homogeneous positive in the tumor cells. Inset: interphase fluorescence in situ hybridization (FISH) analysis using an *IRF4* break apart probe. The cell depicted show one allele with a normal colocalized signal (yellow arrow) and the second allele with a split red and green signals (green and yellow arrows) indicating a rearrangement

LBCL*-IRF4* was introduced as a provisional entity in the WHO 2017 classification under the group of FL [1], and now is upgraded to a definite entity in the 2022 ICC. It remains within the group of FL [2]. The 5th WHO classification [3] also recognizes LBCL-*IRF4* as a definite entity but grouped this disease with large B-cell cell lymphomas, which might be misleading, giving the impression that LBCL-*IRF4* is an aggressive disease. LBCL*-IRF4* is most common in children and young adults and preferentially involves the head and neck region, particularly Waldeyer’s ring but also the intestine [53]. However, it can also occur in adults [54, 55]. It usually presents as a localized disease (stages 1–2) with excellent prognosis, regardless of the growth pattern (see below). Some cases presenting with purely follicular growth patterns, in children, have been reported to achieve complete remission without systemic treatment [56, 57], stressing the indolent nature of the disease. LBCL*-IRF4* is characterized by large B-cells with the expression of at least one GC marker and constitutive expression of MUM1/IRF4, which correlates with *IRF4* translocation (Fig. 2). The growth pattern might be follicular, follicular/diffuse, or diffuse [54]. The cells lack *BCL2-R* or *MYC*-*R*. However, some cases could harbor *BCL6-R* together with *IRF4-R*. FISH for *IRF4-R* is preferred for diagnosis, but cases lacking demonstrable rearrangements should have evidence of either *IGH* or *IGK/IGL* breaks. Some cases can display a cryptic *IRF4* rearrangement leading to a false *IRF4-R*-negative by FISH. In these cases, the detection of *IRF4* mutation may support the diagnosis. In addition, *IRF4-R* can occur as a secondary event in other aggressive B-cell lymphomas in adults, and in this context, the rearrangement is not specific for the diagnosis of LBC*L-IRF4* [54, 55].

## Marginal zone lymphomas

Three types of marginal zone lymphomas (MZL) are currently recognized: splenic MZL (SMZL), extranodal MZL (MALT lymphoma), and primary nodal MZL (NMZL). While these MZL exhibit somewhat overlapping morphologic and phenotypic features, they differ in their clinical presentation and behavior, associations with predisposing conditions, and genetic findings. Each of these conditions therefore represents a distinct clinicopathologic category. Historically, these diagnoses have been challenging due to an absence of sensitive phenotypic or genetic biomarkers. More recently, genomic studies have identified recurrent mutational profiles that, while not diagnostic on their own, can provide helpful ancillary data to support a diagnosis together with other clinical, morphologic, and phenotypic findings.

### Splenic marginal zone lymphoma (SMZL)

SMZL is a small B-cell neoplasm that surrounds and replaces the normal white pulp nodules of the spleen [58–60]. SMZL represents < 2% of all lymphoid neoplasms, shows a median age of 69 years at first diagnosis, and effects males and females equally. Patients present with splenomegaly and involvement of the bone marrow and usually peripheral blood. Anemia and thrombocytopenia may be present. While splenic hilar nodes may be involved, more distant nodal disease is rare and should prompt consideration of an alternate diagnosis.

**Fig. 3:**
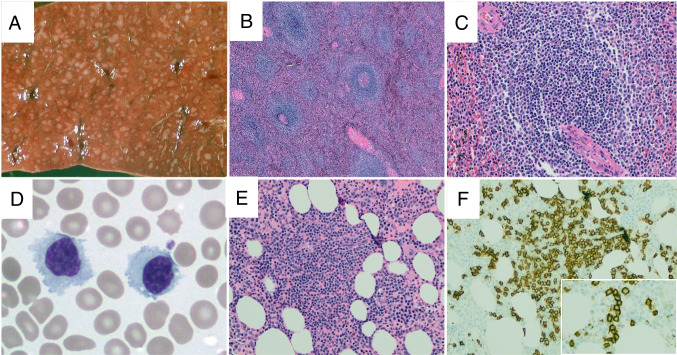
Splenic marginal zone lymphoma. On gross examination, the splenic parenchyma shows increased and expanded white pulp nodules (**A**). On H&E sections, the splenic parenchyma shows increased numbers of white pulp nodules (**B**), which are typically composed of central cores containing small lymphocytes with scant cytoplasm and a peripheral zone of marginal zone cells with more abundant cytoplasm (**C**, H&E). In the peripheral blood, the neoplastic cells are small, often with a somewhat plasmacytoid appearance, and, in some cases, cytoplasmic projections may be seen (**D**, Wright’s stain). In trephine biopsy samples, interstitial lymphoid aggregates are present (**E**, H&E) with numerous small B-cells seen within the aggregate (**F**, CD20). Often, at least focal areas will display an intrasinusoidal growth pattern of small B-cells (inset, **F**)

Establishing a definitive diagnosis of SMZL requires the evaluation of a splenectomy specimen. There is splenomegaly, typically > 400 g and often > 2 kg. The histologic sections show an expansion of white pulp nodules, classically described as having a biphasic appearance with central cores of small lymphocytes with mature chromatin and scant cytoplasm and a peripheral zone of cells with round to slightly irregular nuclei, slightly more open chromatin, and more abundant cytoplasm (Fig. 3). This “classic” biphasic appearance, however, occurs in only half of cases with the remainder showing white pulp nodules composed of monocytoid-appearing cells [61]. Scattered large transformed cells may be seen admixed with monocytoid-appearing small lymphocytes. There is infiltration of the red pulp, often producing small aggregates. In splenic hilar lymph nodes, there is a vaguely nodular proliferation of small, often monocytoid-appearing cells, typically associated with FDC meshworks.

As splenectomy is rarely performed for initial diagnosis in the modern era, most cases are diagnosed via bone marrow biopsy (Fig. 3). In trephine biopsies, SMZL typically infiltrates in an interstitial pattern which may contain distinct aggregates and often exhibits at least a partially intrasinusoidal growth pattern best visualized by immunohistochemistry. The peripheral blood may show lymphocytosis with atypical lymphocytes often having a plasmacytoid appearance. Lymphocytes with cytoplasmic projections, historically termed “villous lymphocytes,” may be seen although this feature is not required for diagnosis [59]. It should be noted that establishing a diagnosis of SMZL from peripheral blood and bone marrow alone, rather than splenic histology, does not allow complete distinction of SMZL from other splenic-based lymphomas such as splenic diffuse red pulp small B-cell lymphoma, although it is not clear that this distinction clinically meaningful.

The neoplastic cells of SMZL generally show a nonspecific phenotype lacking expression of Annexin A1, CD5, CD10, CD23, CD25, CD43, Cyclin D1, and LEF1. CD103 is usually negative. CD5 has been reported in a minority of cases [62, 63]. Recent studies have reported the expression of MNDA and IRTA1 in 25–100% and 0–50% of cases, respectively [64–68]. Using metaphase karyotyping, the most common recurrent abnormality is a deletion of chromosome 7q found in approximately 30% of cases. The t(11;18)(q21;q21) *BIRC3*::*MALT1* translocation and other translocations found in extranodal MZL are absent in SMZL.

NGS studies have identified recurrent mutations in SMZL that, while not sufficiently specific to be used diagnostically in isolation, can provide additional information to help distinguish SMZL from other small B-cell neoplasms in challenging cases. A recent comprehensive genomic study of more than 300 cases of splenectomy-defined SMZL [69] identified two specific molecular subsets of SMZL which together accounted for 91% of SMZL cases. The NNK subset is defined by mutations in the *NOTCH1*, *NFKB*, and *KLF2* pathways which regulate normal marginal zone development. The DMT subset is characterized by mutations in DNA damage repair (e.g., *TP53* and *ATM*) or the MAPK or TLR pathways. The NNK subset was noted to display a less favorable prognosis, especially when accompanied by an immune-suppressive microenvironment as defined by immunohistochemistry.

The differential diagnosis of SMZL is broad and includes most other small B-cell neoplasms, many of which may involve the spleen with a white pulp growth pattern as well as reactive lymphocytosis such as polyclonal B-cell lymphocytosis showing an intrasinusoidal distribution of B lymphocytes resembling marrow involvement by SMZL. In most cases, an accurate diagnosis is accomplished through the use of a standard immunohistochemical/flow cytometric panel, and, in some cases, the molecular assessment of B–cell clonality may be required. A subset of cases of monoclonal B lymphocytosis (MBL) are negative for CD5 and may create a differential diagnosis with SMZL. Importantly, the extent of histologic involvement of the bone marrow does not define a distinction between SMZL and CD5^−^ MBL (so-called non-CLL type MBL or clonal B lymphocytosis of marginal zone origin “BCL-MZ”). In the 2022 ICC [2], the presence of splenomegaly excludes a diagnosis of MBL, and the demonstration of a clonal B-cell population with an appropriate phenotype in the setting of splenomegaly is sufficient for the diagnosis of SMZL.

### Extranodal marginal zone lymphoma (MALT lymphoma)

MALT lymphomas is an extranodal small B-cell neoplasm that essentially recapitulates, to varying extents, normal mucosa-associated lymphoid tissue (MALT), as seen in normal Peyer’s patches or palatine tonsils. MALT lymphomas are generally thought to arise from a background of acquired MALT, in response to various infectious, autoimmune, or other inflammatory stimuli, which leads to an eventual neoplastic transformation. The most common site of involvement is the stomach, but MALT lymphomas may arise at virtually any anatomic location. In the 2022 ICC [2], primary cutaneous cases are separately classified as “primary cutaneous marginal zone lymphoproliferative disorders” due to their very indolent nature (discussed in this issue with other cutaneous lymphoproliferative disorders) while cases arising at all other anatomic sites are classified together under the term of MALT lymphoma.

MALT lymphomas are the most common form of MZL, accounting for 5–8% of all B-cell lymphomas [62, 70]. MALT lymphomas typically occur in adults with a median age at diagnosis in the seventh decade. Both men and women are equally affected overall, although gender predominance may vary at specific anatomic sites. Most cases present with localized (stage IE or IIE) disease, although bone marrow involvement has been reported in 5–10% of cases. Widespread nodal disease is rare.

**Fig. 4:**
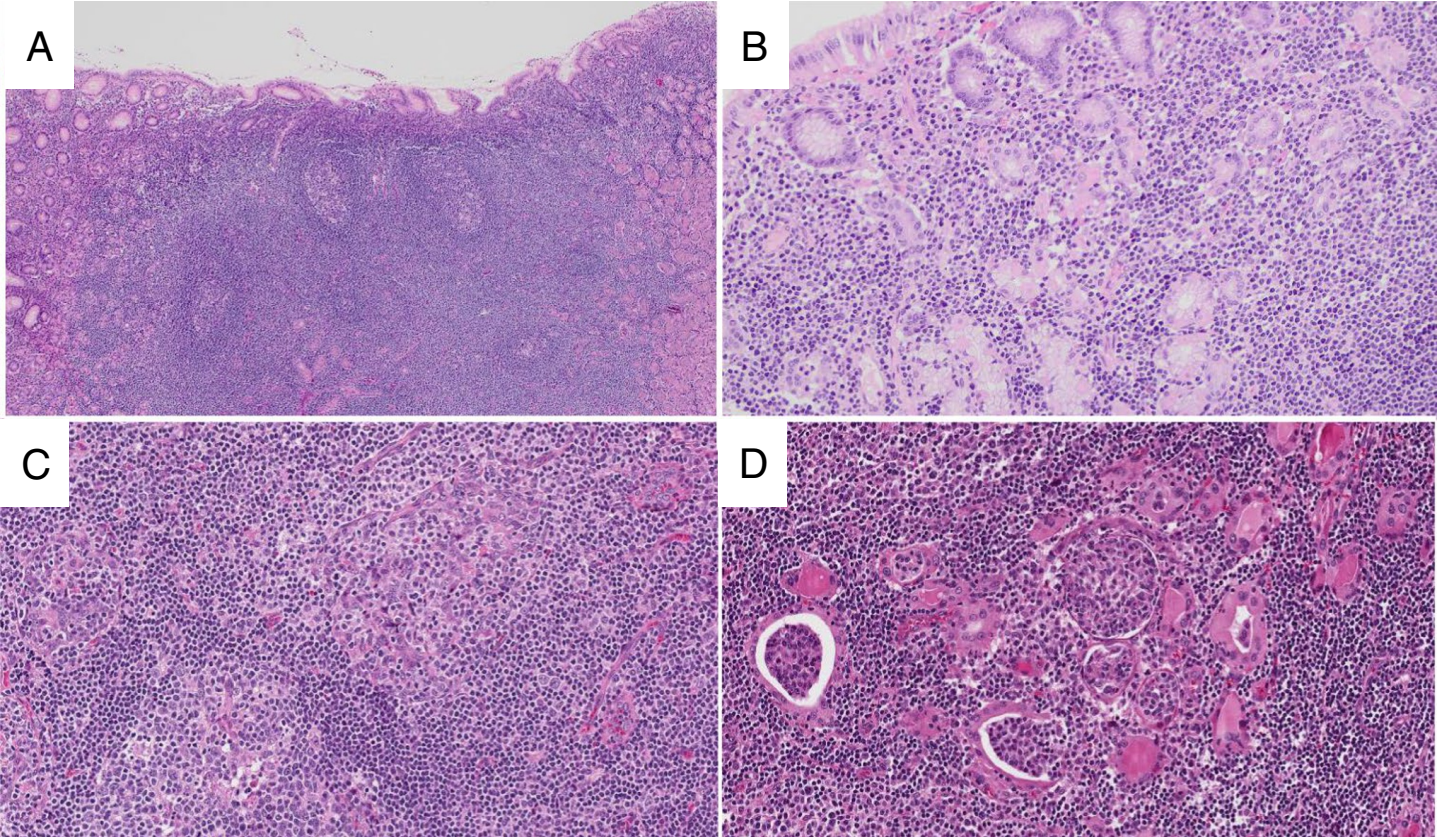
Extranodal marginal zone lymphoma. In gastric MALT lymphoma, the mucosa contains a dense infiltrate of small lymphocytes and germinal centers (**A**, H&E). The small lymphocytes form desctructive lymphoepithelial lesions (**B**, H&E). In this salivary gland MALT lymphoma (**C**, H&E), the lymphoid proliferation contains germinal centers (lower left) and prominent lymphoepithelial lesions (upper right). In thyroid MALT lymphomas (**D**, H&E), lymphoepithelial lesions consist of small lymphocytes within glandular lumens (so-called MALT balls)

The histologic features of MALT lymphoma are generally similar across anatomic sites, although there are some notable site-specific differences. The neoplastic B-cells are small with mature chromatin, round to slightly irregular nuclear contours, and variable amounts of cytoplasm. Overt monocytoid features are typical at some locations, such as the parotid gland, and may be present to a varying extent at other locations (Fig. 4). The tumor cells proliferate around the periphery of GCs, following a marginal distribution external to preserved mantle zones. Diffuse sheets of small neoplastic cells are typically present between GCs. Lymphoepithelial lesions are variable across sites, being present in almost all gastric or parotid gland tumors while they may be inconspicuous or absent in the colon or conjunctiva. The neoplastic cells may invade and colonize GCs, gradually replacing the GC B-cells and creating expanded FDC meshworks.

While the morphologic findings in MALT lymphoma are characteristic, definitive diagnosis requires correlation with immunophenotypic studies in order to rule out a reactive lymphoid hyperplasia and other small B-cell neoplasms which may also present at extranodal locations [62, 71]. The neoplastic cells are positive for pan-B-cell antigens. They are generally negative for CD5, CD10, CD23, LEF1, Cyclin D1, and SOX11, although rare CD5 or CD10-positive cases have been described. CD43 is expressed in ~ 50% of cases, and this aberrant phenotype can assist in the differential diagnosis with a reactive process, although it is also found in a proportion of other small B-cell tumors. If fresh tissue is available, flow cytometric analysis may be helpful to evaluate light chain restriction in B-cells. In situ hybridization and immunohistochemical stains can help identify light chain restriction in cases with plasmacytic differentiation, but such stains are often insufficiently sensitive to evaluate B-cell clonality. More recently described ultrasensitive in situ hybridization stains offer the ability to routinely assess both B-cell and plasma cell clonality [72].

Recent years have seen efforts to identify biomarkers specific to MZL. MNDA expression has been reported in 61–95% of MALT lymphomas, and the presence of this marker can help distinguish MALT from follicular lymphoma which only rarely expresses MNDA. IRTA1 has been described in 52–93% of MALT lymphomas and appears to be only rarely expressed in non-MZL [64–68]. IRTA1 therefore may serve as a specific, but not especially sensitive, marker of MZL.

Immunoglobulin heavy chains are clonally rearranged in MALT lymphoma, with evidence of somatic hypermutation in most cases, consistent with a post-GC cell of origin. Within specific anatomic sites, there is often biased usage of specific *IGH* variable gene families, supporting the concept of a common predisposing antigenic response which may precede the development of an overt lymphoma. Recurrent translocations have been identified in MALT lymphoma (Table 2), all of which lead to dysregulation of the NFKB pathway. The identification of these translocations can be diagnostically useful, as they do not occur in other small B-cell neoplasms, and, in some cases, they may directly impact prognosis or impact therapeutic decision-making. For example, the *BIRC3*::*MALT1* fusion, found in approximately 25% of gastric MALT lymphomas, is associated with disseminated disease and lack of response to antibiotic therapy. An increasing number of NGS studies have identified recurrently mutated genes in MALT lymphoma (Table 2). The most frequently mutated genes may vary across anatomic sites, again reflecting site-specific differences in pathogenesis [73–75]. While most MALT lymphomas may be diagnosed on the basis of morphology and phenotype alone, NGS studies may be helpful in the differential diagnosis with other small B-cell disorders in selected cases. Specifically, *MYD88* L265P mutations are found in only a small number of MALT lymphomas and *CXCR4* mutations have only rarely been described, assisting in the differential diagnosis with an extranodal lymphoplasmacytic lymphoma.

**Table 2:** Molecular/cytogenetic abnormalities in extranodal MALT lymphomas

Site	Translocations/trisomies	Mutations
Stomach	*BIRC3::MALT1* (6–23%) *IGH::MALT1* (1–5%) + 3 (11%) + 18 (6%)	*NOTCH1* (17%) *NF1* (16%) *TNFAIP3* (15%) *ATM* (13%) *TRAF3* (13%)
Occular adnexa/orbit	*IGH::MALT1* (0–25%) *FOXP1::IGH* (0–20%) *BIRC3::MALT1* (0–10%) + 3 (38%) + 18 (13%)	*TNFAIP3* (39%) *KMT2D* (15%) *CREBBP* (10%) *LRP1B* (10%) *MYD88* (10%)
Salivary gland	*IGH::MALT1* (0–16%) *BIRC3::MALT1* (0–5%) *BCL10::IGH* (0–2%) + 3 (55%) + 18 (19%)	*TBL1XR1* (24%) *GRP34* (16%) *NOTCH2* (11%) *SPEN* (11%) *KMT2C* (11%)
Lung	*BIRC3::MALT1* (31–53%) *IGH::MALT1* (6–10%) *BCL10::IGH* (2–7%) + 3 (20%) + 18 (4%)	*KMT2D* (25%) *TNFAIP3* (18%) *PRDM1* (12%) *NOTCH1* (12%) *EP300* (11%)
Thyroid	*FOXP1::IGH* (0–50%) *BIRC3::MALT1* (0–17%) + 3 (17%)	*TET2* (61%) *TNFRSF14* (44%) *PIK3CD* (23%) *SPEN* (17%) *CREBBP* (8%)

Summarized from Vela et al. [73], Streubel et al. [74], and Remstein et al. [75]

### Nodal marginal zone lymphoma (NMZL)

NMZL is a primary nodal small B-cell neoplasm that resembles secondary lymph node involvement by SMZL or extranodal MALT lymphoma. NMZL is the least common form of MZL and makes up only 1–2% of all non-Hodgkin lymphomas. The median age at presentation is in the 6th decade [76].

**Fig. 5:**
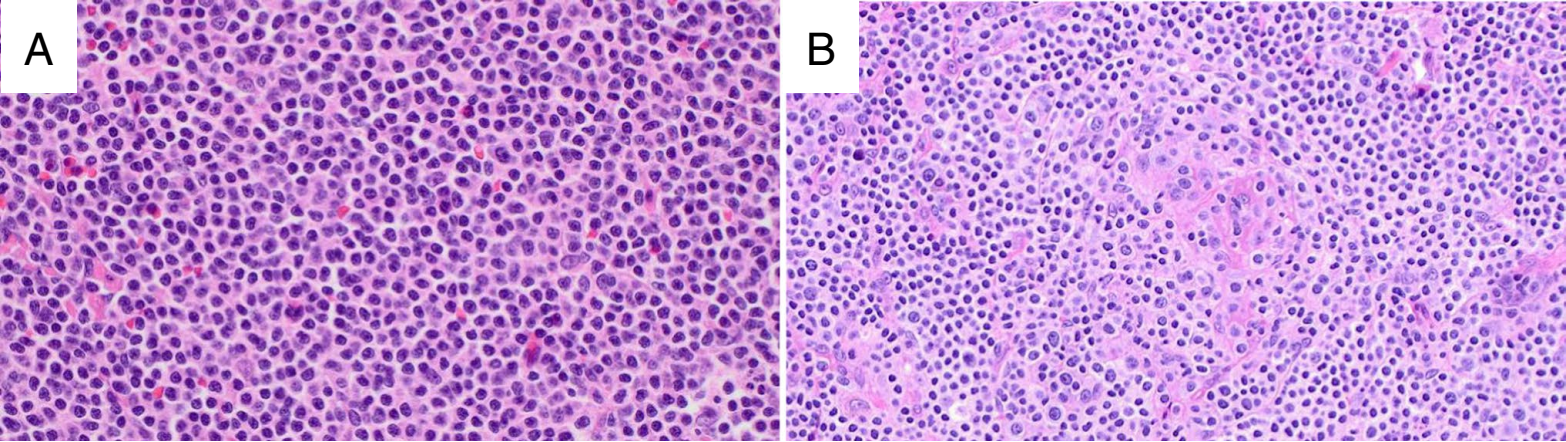
Nodal marginal zone lymphoma. The histologic appearance of NMZL is variable: Panel **A** (H&E) shows a NMZL with a diffuse growth pattern and predominantly small lymphocytes with little cytoplasm, while the NMZL in panel **B** (H&E) displays abundant pale cytoplasm and a nodular appearance with colonized germinal centers

The morphologic appearance of NMZL is quite heterogeneous, with the neoplastic cells consisting of small lymphocytes, monocytoid-appearing cells, and plasmacytoid cells in varying proportions (Fig. 5). Architecturally, there is often at least a partially nodular appearance where the neoplastic cells surround, invade, and colonize residual GC. Other cases exhibit a purely diffuse growth pattern. Overt plasmacytic differentiation may be present, leading to a differential diagnosis with nodal lymphoplasmacytic lymphoma.

Immunophenotypic studies show the expression of pan B-cell markers. CD5 and CD10 are characteristically absent, although CD5 has been reported in 10–20% of cases. CD43 may be coexpressed in 20–75% of cases, and IRTA1 and MNDA have been reported in 54–75% and 43–73%, respectively [64–68]. CD23 expression has been reported in up to 30% of cases [77], although recent data suggest these cases may be more similar to *BCL2*-*R* negative CD23 + FCL rather than NMZL [33].

Relatively little genetic data is available on NMZL, but the literature to date suggests cases diagnosed as NMZL are biologically heterogeneous [78, 79]. Trisomies of chromosomes 3 and 18 are recurrent, but seen in under 20% of cases. The most highly recurrent mutations include *MLL2* (*KMT2D*), *PTPRD*, *NOTCH2*, and *KLF2*, reported in approximately 20–30% of cases. A large number of genes have been reported to be recurrently mutated but in 10% or fewer cases, and no single gene is known to be mutated in the majority of NMZL. The results suggest that NMZL, as currently defined by diagnostic criteria, is biologically heterogeneous, and additional studies will be required in an effort to identify one or more homogeneous entities within this group of cases. The 2017 WHO and 2022 ICC recognized pediatric form pNMZL as a provisional entity [1, 3]. Recent data, however, has suggested that pNMZL is genetically similar to PTFL [48, 80] and it has been suggested that cases previously termed pNMZL may be better designated as PTFL with marginal zone differentiation.

## Conclusion

Recent advances in the biology of FL and MZL, especially through broad-based genomic profiling, have provided a deeper understanding of FL and MZL and greater recognition of unique clinicopathologically defined subtypes. Alternative forms of FL, especially those negative for *BCL2-R*, are biologically and/or clinically distinct from conventional FL and should be distinguished. Rendering a correct diagnosis of specific FLs and MZLs requires knowledge of the clinical context including the site of involvement, age, and clinical presentation as well as thorough phenotypic studies. In difficult cases, the use of ancillary tests such as NGS and FISH testing could be useful to accurately diagnose these FL variants and various MZL subtypes.
